# Superior performance and high service stability for GeTe-based thermoelectric compounds

**DOI:** 10.1093/nsr/nwz052

**Published:** 2019-04-10

**Authors:** Tong Xing, Qingfeng Song, Pengfei Qiu, Qihao Zhang, Xugui Xia, Jincheng Liao, Ruiheng Liu, Hui Huang, Jiong Yang, Shengqiang Bai, Dudi Ren, Xun Shi, Lidong Chen

**Affiliations:** 1 State Key Laboratory of High Performance Ceramics and Superfine Microstructure, Shanghai Institute of Ceramics, Chinese Academy of Sciences, Shanghai 200050, China; 2 Center of Materials Science and Optoelectronics Engineering, University of Chinese Academy of Sciences, Beijing 100049, China; 3 Materials Genome Institute, Shanghai University, Shanghai 200444, China

**Keywords:** thermoelectrics, phase transition, thermal expansion, service stability, power generation application

## Abstract

GeTe-based compounds have been intensively studied recently due to their superior thermoelectric performance, but their real applications are still limited so far due to the drastic volume variation that occurs during the rhombohedral–cubic phase transition, which may break the material or the material/electrode interface during service. Here, superior performance and high service stability for GeTe-based thermoelectric compounds are achieved by co-doping Mg and Sb into GeTe. The linear coefficient of thermal expansion before phase transition is greatly improved to match that after phase transition, yielding smooth volume variation around the phase transition temperature. Likewise, co-doping (Mg, Sb) in GeTe successfully tunes the carrier concentration to the optimal range and effectively suppresses the lattice thermal conductivity. A peak *zT* of 1.84 at 800 K and an average *zT* of 1.2 in 300–800 K have been achieved in Ge_0.85_Mg_0.05_Sb_0.1_Te. Finally, a Ni/Ti/Ge_0.85_Mg_0.05_Sb_0.1_Te thermoelectric uni-leg is fabricated and tested, showing quite good service stability even after 450 thermal cycles between 473 K and 800 K. This study will accelerate the application of GeTe-based compounds for power generation in the mid-temperature range.

## INTRODUCTION

The International Energy Agency's *Global Energy* & *CO_2_ Report* (2017) showed that global energy demand grew by 2.1% in 2017, more than twice as much as the increase in 2016. Meanwhile, global energy-related CO_2_ emission grew by 1.4% in 2017, a resumption of growth after three years of stabilized global emissions [1]. Such huge energy demands and severe CO_2_ emissions impose a pressing need to use energy more efficiently.

As a sustainable and eco-friendly energy conversion technology, thermoelectric (TE) technology has drawn increasing attention from both academic and industrial communities [2]. It can be potentially used to convert the waste heat from vehicle exhausts or plants directly into useful electricity, providing an alternative way to more efficiently utilize fossil energy [3]. Large-scale application of TE technology requires highly efficient and reliable TE devices [4,5]. The efficiency of a TE device is related to the material's TE figure of merit *zT* = *S*^2^*σT*/(*κ_e_* + *κ_L_*), where *S* is the Seebeck coefficient, *σ* is the electrical conductivity, *κ_L_* is the lattice thermal conductivity, *κ_e_* is the carrier thermal conductivity, and *T* is the absolute temperature. Thus, developing TE materials with high *zT* and good service stability is the foremost task in thermoelectrics.

**Figure 1.**
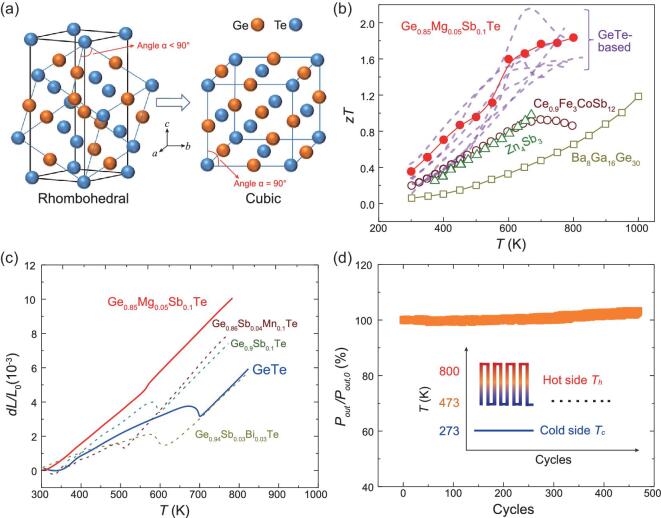
(a) Crystal structures of the low-temperature rhombohedral phase and high-temperature cubic phase in GeTe. (b) TE figure of merit (*zT*) for Ge_0.85_Mg_0.05_Sb_0.1_Te and some typical p-type TE materials. The dashed lines represent the data for GeTe-based compounds reported in the literature. (c) Temperature dependence of relative length variation (*dL*/*L_0_*) for GeTe and Ge_0.85_Mg_0.05_Sb_0.1_Te. The values on the *dL*/*L_0_* curves represent the linear coefficients of thermal expansion (CTE) in the specific temperature range. The dashed lines represent the data for Ge_0.9_Sb_0.1_Te, Ge_0.94_Sb_0.03_Bi_0.03_Te, and Ge_0.86_Sb_0.04_Mn_0.1_Te respectively. (d) Variation of relative power output (*P*_out_*/P*_out_*_,0_*) of a Ni/Ti/Ge_0.85_Mg_0.05_Sb_0.1_Te TE leg during a thermal cycling test. The inset shows a schematic map of the thermal cycling test. The hot side temperature is cycled from 473 K to 800 K. The cold side temperature is fixed at 300 K. The data are collected when the hot side temperature of the uni-leg is 800 K.

Over the past two decades, the TE community has witnessed unprecedented success in improving the *zT* of classical TE materials and in the discovery of many types of novel TE materials [6–16]. As a rapidly growing class of TE materials, GeTe-based compounds are very special and were to give high TE performance as early as the 1960s, but only recently has more attention been paid to them [17–19]. GeTe is a p-type narrow band-gap semiconductor crystallizing with a rhombohedral structure (*R3m*) at room temperature (Fig. 1a). Around 700 K, the rhombohedral *R3m* structure undergoes a ferroelectric structure transition and converts to the high-temperature cubic *Fm*${\rm{\bar{3}}}$*m* structure (Fig. 1a) [20]. Due to the presence of severe intrinsic Ge vacancy inside the lattice, GeTe has a very high carrier concentration in the order of 10^21^ cm^−3^ at room temperature yielding very low *S* (∼ 30 *μ*V K^−1^) and high ${\kappa _e}$ (∼ 5 W  m^−1^  K^−1^) [21–23]. Thus, most of the studies for GeTe focus on reducing the over-high carrier concentration and pushing it to the optimal range. Along this route, many high-performance p-type GeTe-based compounds, such as Ge_0.9_Sb_0.1_Te_0.9_Se_0.05_S_0.05_ with a *zT* of 2.1 at 630 K [24], Ge_0.89_Sb_0.1_In_0.01_Te with a *zT* of 2.3 at 750 K [25], Ge_0.86_Mn_0.1_Sb_0.04_Te with a *zT* of 1.61 at 823 K [26], Ge_0.86_Mn_0.1_Bi_0.04_Te with a *zT* of 1.5 at 773 K [27], (GeTe)_0.73_(PbSe)_0.27_ with a *zT* of 2.2 at 800 K [28], and Ge_0.87_Pb_0.13_Te with a *zT* of 2.2 at 700 K [29], have been reported. These high *zT* values are much superior to those of classic mid-temperature p-type TE materials, such as CeFe_3_CoSb_12_ [30], Ba_8_Ga_16_Ge_30_ [31], and Zn_4_Sb_3_ [32], which have maximum *zT* values around unity (Fig. 1b).

Despite the superior *zT* values, the real application for GeTe-based compounds is greatly limited by the drastic volume variation occurring during the rhombohedral–cubic phase transition. As shown in Fig. 1c, an obvious discontinuity in the temperature dependence of the relative length variation (*dL*/*L_0_*) is observed for GeTe around 700 K. The measured linear coefficient of thermal expansion (CTE) before the phase transition is only 11.2 × 10^−6^ K^−1^, while it increases to as high as 23.4 × 10^−6^ K^−1^ after the phase transition. Moreover, abnormal negative CTE appears during the phase transition range. Similar characteristics have also been observed in many element-doped GeTe compounds, such as Sb-doped GeTe, (Bi, Sb) co-doped GeTe, and (Mn, Sb) co-doped GeTe (Fig. 1c). The drastic volume variation caused by such huge CTE mismatches makes it easy to break GeTe-based materials when they experience frequent thermal cycles due to large thermal stress. This is the possible reason why GeTe-based devices are currently absent although they possess excellent TE performance.

In this study, Mg and Sb are co-doped in GeTe to successfully tune the carrier concentration to the optimal range and effectively suppress the lattice thermal conductivity, leading to a maximum *zT* of 1.84 at 800 K and an average *zT* of 1.2 at 300–800 K in Ge_0.85_Mg_0.05_Sb_0.1_Te (Fig. 1b). Furthermore, the CTE of the low-temperature phase is obviously enhanced to match the high-temperature phase (Fig. 1c) for good service stability, which is well demonstrated in a Ni/Ti/Ge_0.85_Mg_0.05_Sb_0.1_Te TE uni-leg that has quite stable power output even after 450 thermal cycles between 473 K and 800 K (Fig. 1d).

## RESULTS AND DISCUSSION

Volume expansion with increasing temperature is determined by the increased kinetic energy of composed atoms [33]. The crystal structure of the GeTe low-temperature rhombohedral phase can be viewed as a slightly distorted rock-salt lattice along the (111) direction [34], which has a pseudo-cubic lattice parameter *a_c_* and a pseudo-cubic angle *α*. Both *a_c_* and *α* can be related to the lattice constants (*a* and *c*) of the hexagonal unit cell in GeTe via the relations *a* = 2*a_c_* sin (*α*/2) and *c* = *a_c_* (3+6cos*α*)^1/1^ [35]. The distortion degree is reflected by the deviation of the pseudo-cubic angle *α* from 90° (Fig. 1a). In GeTe, *α* is about 88.28° at 300 K, while it is 90° above the phase transition temperature. Such small deviation in *α* is believed to be one of the origins of the CTE mismatch between the low- and high-temperature phases. If the *α* of the rhombohedral phase is close to 90°, the CTE of the low- and high-temperature phases should be quite similar and thus is suitable to be used to fabricate stable TE devices.

**Figure 2.**
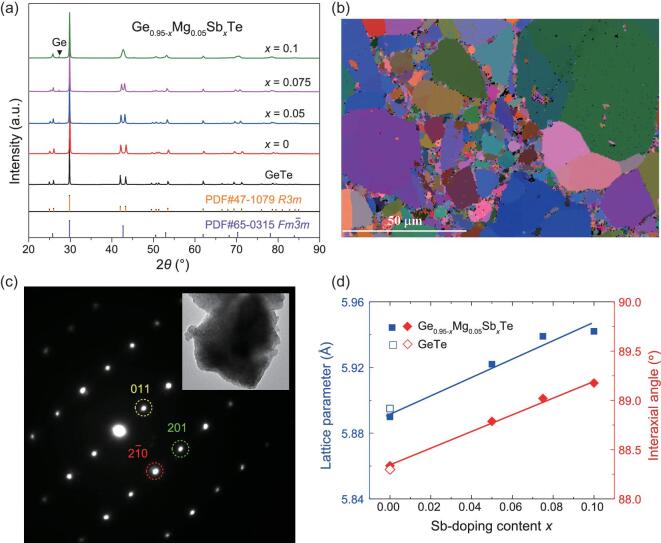
(a) Powder X-ray patterns of Ge_0.95-_*_x_*Mg_0.05_Sb*_x_*Te (*x* = 0, 0.05, 0.075, and 0.1). (b) Electron backscatter diffraction (EBSD) characterization performed on the sintered bulk Ge_0.85_Mg_0.05_Sb_0.1_Te sample. (c) Selected area electron diffraction (SAED) patterns along the zone axis of <1${\rm{\bar{1}}}$0> performed on the Ge_0.85_Mg_0.05_Sb_0.1_Te particle shown in the inset. (d) Refined pseudo-cubic lattice parameter (*a_c_*) and pseudo-cubic angle (*α*) as a function of Sb-doping content. The solid lines in (d) are guides for the eyes.

Here we choose Mg and Sb co-dopants to tune the lattice parameters and TE properties of GeTe-based compounds. Figure 2a shows the room-temperature powder X-ray diffraction (PXRD) patterns for Ge_0.95-_*_x_*Mg_0.05_Sb*_x_*Te (*x* = 0, 0.05, 0.075, and 0.1). The main diffraction peaks for Ge_0.95_Mg_0.05_Te can be well identified as belonging to the rhombohedral structure (*R3m*). A very tiny amount of Ge precipitates is observed, which is observed commonly in GeTe-based compounds [28,36,37]. The energy dispersive spectroscopy (EDS) elemental mapping for Ge_0.95_Mg_0.05_Te shows that there is no obvious Mg-rich phase (Supplementary Fig. S1), confirming that all Mg atoms enter the lattice of GeTe. The actual chemical composition of Ge_0.95_Mg_0.05_Te characterized by electron probe microanalysis is listed in Table 1; this is almost the same as the nominal compositions. With increasing Sb-doping content, the double peaks [(024) and (220)] in 2*θ* around 41–45° gradually approach each other. When the Sb-doping content *x* = 0.1, the double peaks almost merge together, suggesting that the pseudo-cubic angle *α* should be close to 90°. Likewise, the EDS elemental mapping proves that there is no obvious Sb-rich or Mg-rich phase in the Ge_0.85_Mg_0.05_Sb_0.1_Te matrix (Supplementary Fig. S2). The electron backscatter diffraction (EBSD) characterization performed on the sintered bulk Ge_0.85_Mg_0.05_Sb_0.1_Te sample shows that the grain size has a quite wide distribution in the range of 1–50 μm (Fig. 2b).

**Table 1.** The actual chemical and nominal compositions of Ge_0.95_Mg_0.05_Te and Ge_0.9_Mg_0.05_Sb_0.05_Te characterized by electron probe microanalysis.

		
Ge_0.95-_*_x_*Mg_0.05_Sb*_x_*Te	*x* = 0	*x* = 0.05
		
Nominal composition	Ge_0.95_Mg_0.05_Te	Ge_0.9_Mg_0.05_Sb_0.05_Te
Actual composition	Ge_0.89(5)_Mg_0.05(1)_Te_1.05(4)_	Ge_0.81(4)_Mg_0.05(4)_Sb_0.05(2)_Te_1.09(1)_

Figure 2c shows the selected area electron diffraction (SAED) patterns along the zone axis of <1${\rm{\bar{1}}}$0> performed on Ge_0.85_Mg_0.05_Sb_0.1_Te, which can be well identified as the rhombohedral structure. Rietveld refinement based on room-temperature X-ray data is performed to obtain the pseudo-cubic lattice parameter (*a*_c_) and pseudo-cubic angle (*α*). The details can be found in Supplementary Fig. S3. As shown in Fig. 2d, doping Mg into GeTe scarcely alters *a*_c_ and *α*, which is reasonable considering the similar ionic radii between Mg^2+^ (0.72 ${\rm{{\mathring{\rm A}}}}$) and Ge^2+^ (0.73 ${\rm{{\mathring{\rm A}}}}$). In contrast, with increasing Sb-doping content, both the *a*_c_ and *α* gradually increase. For Ge_0.85_Mg_0.05_Sb_0.1_Te, *α* is already enlarged to 89.18°. Supplementary Fig. S4 shows the heat-flow curves for Ge_0.95-_*_x_*Mg_0.05_Sb*_x_*Te (*x* = 0, 0.05, 0.075, and 0.1) measured by differential scanning calorimetry (DSC). All samples have an endothermic peak around 500–700 K, indicating that the rhombohedral phase converts to the cubic phase in this temperature range.

Because the pseudo-cubic angles *α* in the (Mg, Sb) co-doped samples are close to 90°, the CTE for the rhombohedral phase is also expected to be close to that for the high-temperature cubic phase. This is confirmed by the measured relative length variation *dL*/*L_0_* and CTE for the (Mg, Sb) co-doped materials (Fig. 1c, Fig. S5 and Table 2). With increasing Sb-doping content, the CTE before the phase transition gradually increases from 11.2 × 10^−6^ K^−1^ for GeTe to 19.2 × 10^−6^ K^−1^ for Ge_0.85_Mg_0.05_Sb_0.1_Te. The latter value is quite close to that after the phase transition (23.4 × 10^−6^ K^−1^). In particular, the abnormal volume contraction during the phase transition range almost disappears for Ge_0.85_Mg_0.05_Sb_0.1_Te. As shown in Fig. 1c, the relative length variation *dL*/*L_0_* for Ge_0.85_Mg_0.05_Sb_0.1_Te changes quite smoothly in the phase transition temperature range, which is beneficial for achieving high stability during service. Since solely doping Mg or Sb into GeTe cannot eliminate such negative CTE (Fig. 1c), the disappearing abnormal volume contraction is believed to be due to the coupling effect of the Mg and Sb dopants.

**Table 2.** Room-temperature physical properties of Ge_0.95-_*_x_*Mg_0.05_Sb*_x_*Te (*x* = 0, 0.05, 0.075, and 0.1). The data for GeTe are also included.

Ge_0.95-_*_x_*Mg_0.05_Sb*_x_*Te		*x* = 0	*x* = 0.05	*x* = 0.075	*x* = 0.1	GeTe
		
CTE (10^−6^ K^−1^)	*R3m*	16.6	17.4	18.5	19.2	11.2
	*Fm* $\bar{3}$ *m*	23.5	24.8	24.7	23.5	23.4
*σ* (10^4^ S m^−1^)		44.4	18.0	9.1	3.7	76.3
*S* (μV K^−1^)		35.5	76.5	99.9	196.4	35.7
*n* (10^20^ cm^−3^)		12.6	4.5	2.9	2.2	7.5
m* (*m_e_*)		2.04	2.23	2.22	4.77	1.45
*μ* (cm^2^ V^−1^ s^−1^)		23.8	25.6	25.7	11.8	87.4
*μ_W_* (cm^2^ V^−1^ s^−1^)		69.3	85.9	84.9	123.2	152.6
*ρ*(g cm^−3^)		5.92	5.97	5.94	6.10	6.14

**Figure 3.**
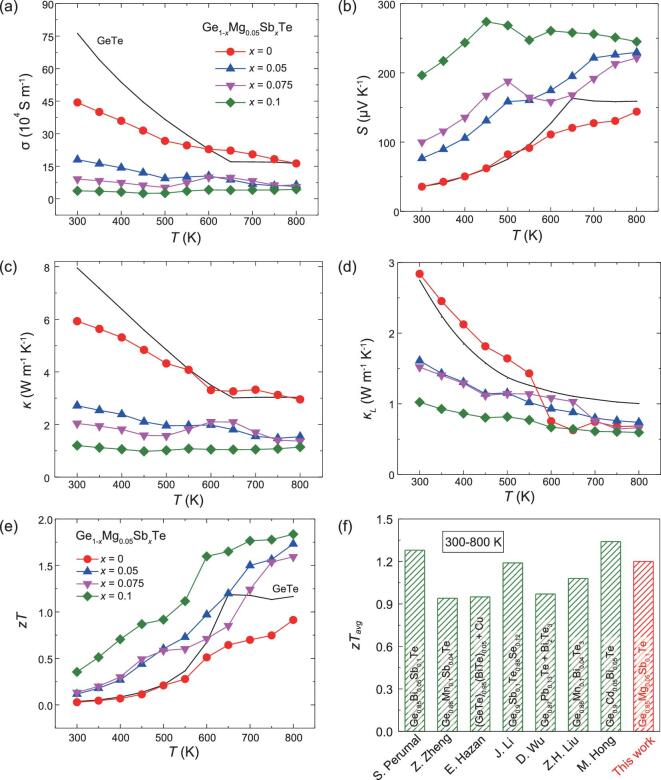
Temperature dependences of (a) electrical conductivity (*σ*), (b) Seebeck coefficient (*S*), (c) thermal conductivity (*κ*), (d) lattice thermal conductivity (*κ_L_*), and (e) TE figure of merit (*zT*) for Ge_0.95-_*_x_*Mg_0.05_Sb*_x_*Te (*x* = 0, 0.05, 0.075, and 0.1). The solid lines represent the data for GeTe. (f) Comparison of the average TE figure of merit (*zT*_avg_) at 300–800 K for several GeTe-based compounds and Ge_0.85_Mg_0.05_Sb_0.1_Te in this work [26,27,38–42].

Figure 3 shows the measured TE properties for Ge_0.95-_*_x_*Mg_0.05_Sb*_x_*Te (*x* = 0, 0.05, 0.075, and 0.1). The data for GeTe are included for comparison. All samples possess positive *S* throughout the entire measured temperature range, indicating that holes are the dominated carriers. Doping Mg into GeTe only slightly changes the *S* but significantly lowers the *σ*. At 300 K, the *σ* for Ge_0.95_Mg_0.05_Te is 4.4 × 10^5^ S m^−1^, about 40% of that for GeTe. However, like GeTe, Ge_0.95_Mg_0.05_Te still shows a typical highly degenerate semiconducting transport behavior with *σ* decreasing with increasing temperature. Compared with the Mg-doping, the Sb-doping in Ge_0.95_Mg_0.05_Te affects the electrical transport substantially (see Fig. 3a and b). With increasing Sb-doping content from *x* = 0 to *x* = 0.1, the *σ* is gradually decreased. The *σ* for Ge_0.85_Mg_0.05_Sb_0.1_Te is only 3.7 × 10^4^ S m^−1^ at 300 K, about one order of magnitude lower than that for Ge_0.95_Mg_0.05_Te. Likewise, the *S* gradually increases with increasing Sb-doping content in the entire temperature range. The *S* for Ge_0.85_Mg_0.05_Sb_0.1_Te is 196.4 μV K^−1^ at 300 K, about six times that for Ge_0.95_Mg_0.05_Te. However, at temperatures above 600 K, the increment of *S* cannot fully compensate for the negative effect of the decrease of *σ* on the electrical transport properties. Thus, as shown in Supplementary Fig. S6, the power factor *PF* (= *S*^2^*σ*) for the (Mg, Sb) co-doped GeTe samples above 600 K is lower than that for GeTe.

Figure 3d shows the total thermal conductivity (*κ*) for Ge_0.95-_*_x_*Mg_0.05_Sb*_x_*Te (*x* = 0, 0.05, 0.075, and 0.1). All samples possess quite low *κ* values as compared with GeTe. The *κ* for Ge_0.85_Mg_0.05_Sb_0.1_Te is only about 1.2 W m^−1^ K^−1^ at 300 K, about one-eighth of that for GeTe and one-sixth of that for Ge_0.95_Mg_0.05_Te. Such greatly lowered *κ* is a result of two factors. One factor is the reduced carrier contribution in the thermal transport. Supplementary Fig. S7 shows the calculated carrier thermal conductivity *κ_e_* for Ge_0.95-_*_x_*Mg_0.05_Sb*_x_*Te (*x* = 0, 0.05, 0.075, and 0.1) based on the Wiedemann–Franz law (*κ_e_* = *L*_0_*Tσ*, where the Lorenz number *L*_0_ is estimated based on the single parabolic band model with the value shown in Supplementary Fig. S8). Clearly, the (Mg, Sb) co-doping in GeTe greatly suppresses *κ_e_* by lowering *σ*. In addition, due to the atomic size and mass mismatch among Ge, Mg, and Sb, doping Mg and Sb at Ge sites also introduces additional mass and strain field fluctuations to strongly scatter heat-carrying phonons to lower the lattice thermal conductivity *κ_L_*. Figure 3d shows the calculated *κ_L_* for Ge_0.95-_*_x_*Mg_0.05_Sb*_x_*Te (*x* = 0, 0.05, 0.075, and 0.1) by subtracting *κ_e_* from the total *κ*. Doping Mg and Sb into GeTe suppresses *κ_L_* in the entire measured temperature range. The *κ_L_* for Ge_0.85_Mg_0.05_Sb_0.1_Te is only 1.0 W m^−1^ K^−1^ at 300 K, about one-third of that for GeTe. Combining the measured *S, σ*, and *κ*, the TE figure of merit *zT* (= *S*^2^*σT/κ*) for Ge_0.95-x_Mg_0.05_Sb*_x_*Te is calculated and shown in Fig. 3e. As a result of the significantly improved *S* and lowered *κ*, the *zT* values of the (Mg, Sb) co-doped GeTe samples are obviously enhanced. The *zT* for Ge_0.85_Mg_0.05_Sb_0.1_Te is 1.84 at 800 K, which is comparable with the best results in GeTe-based compounds reported previously. Moreover, the average figure of merit *zT* over the temperature range (300–800 K) for Ge_0.85_Mg_0.05_Sb_0.1_Te is 1.2 (Fig. 3f), which is among the top values reported in this temperature range [26,27,38–42].

**Figure 4.**
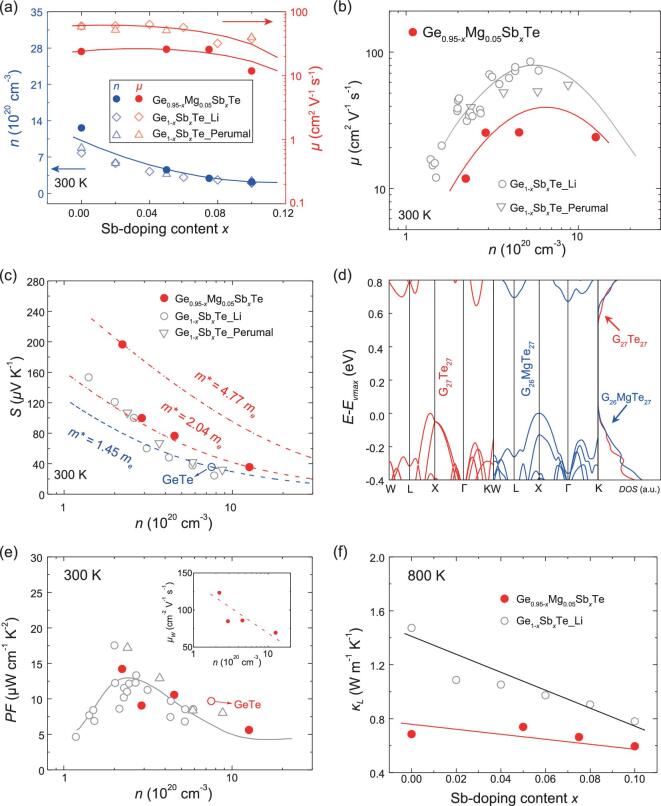
(a) Sb-doping content (*x*) dependences of carrier concentration (*n*) and carrier mobility (*μ*) for Ge_0.95-_*_x_*Mg_0.05_Sb*_x_*Te at 300 K. (b) Relationship between *n* and *μ* at 300 K. (c) Seebeck coefficient (*S*) versus *n* for Ge_0.95-_*_x_*Mg_0.05_Sb*_x_*Te at 300 K. The dashed lines represent the calculated Pisarenko curves with different density-of-state effective masses (m*) predicted by the single parabolic band model. (d) Calculated band structure and density-of-state near the Fermi level for both the undoped GeTe and Mg-doped GeTe rhombohedral phase. (e) Power factor (*PF*) versus *n* at 300 K. The inset shows the weighted mobility (*μ_W_*) as a function of *n*. (f) Lattice thermal conductivity as a function of Sb-doping content *x* at 800 K. The data for the Ge_1-_*_x_*Sb*_x_*Te samples are included in (a–c) and (e, f) for comparison [40,44]. The solid lines in (a, b) and (e, f) are guides for the eyes.

The reduced carrier concentration is one of the main reasons for the enhanced *zT* values in the present (Mg, Sb) co-doped GeTe. Figure 4a shows the measured Hall carrier concentration (*n*) for these samples at room temperature. Due to the presence of severe Ge vacancies inside the lattice, the *n* for the undoped GeTe is as high as 7.5 × 10^20^ cm^−3^, which significantly deviates from the optimal value (around 2 × 10^20^ cm^−3^) for good electrical transport [43]. Although the valence state of Mg is +2 that is identical to that of the host atom Ge, doping Mg into GeTe further increases the *n* to 1.3 × 10^21^ cm^−3^. Similar phenomenon has been also observed in Mn-doped GeTe, in which Mn also adopts a +2 valence state [26]. The increased *n* should be attributed to the increased lattice defects such as Ge vacancies inside the lattice. On the other hand, differing from Mg and Mn, Sb behaves as an electron donor in GeTe. As shown in Fig. 4a, the *n* gradually decreases with increasing Sb-doping content. When the Sb-doping content *x* = 0.1, *n* is reduced to as low as 2.2 × 10^20^ cm^−3^ at 300 K, about one-sixth of that for Ge_0.95_Mg_0.05_Te. For comparison, the *n* data for the Sb single-doped GeTe samples are also included in Fig. 4a [40,44]. Interestingly, with the same Sb-doping content, the Ge_0.95-_*_x_*Mg_0.05_Sb*_x_*Te sample possesses almost the same *n* as that of Ge_1-_*_x_*Sb*_x_*Te, indicating that Mg does not yield extra carriers when it coexists with Sb in GeTe. However, Fig. 4a shows that Mg obviously lowers the carrier mobility (*μ*). With the same Sb-doping content, the Ge_0.95-_*_x_*Mg_0.05_Sb*_x_*Te sample possesses lower *μ* than that for the Ge_1-_*_x_*Sb*_x_*Te samples. The reduced *μ* is responsible for the much-lowered electrical conductivity in the Mg-including GeTe samples (Fig. 3a).

The relationship between *n* and *μ* for Ge_0.95-_*_x_*Mg_0.05_Sb*_x_*Te can be more clearly illustrated in Fig. 4b. The *μ* values for the Ge_0.95-_*_x_*Mg_0.05_Sb*_x_*Te samples obey a similar variation trend to those for the Ge_1-_*_x_*Sb*_x_*Te samples [40,44]. Nevertheless, in a similar *n* range, the *μ* values for the Ge_0.95-_*_x_*Mg_0.05_Sb*_x_*Te samples are much lower than those for the Ge_1-x_Sb*_x_*Te samples, which should be due to the additional defect scattering introduced by Mg dopants at the Ge sites.

Figure 4c plots *S* versus *n* for Ge_0.95-_*_x_*Mg_0.05_Sb*_x_*Te. *S* increases with decreasing *n*. By using a single parabolic band model and assuming that scattering is dominated by acoustic phonons, the theoretical Pisarenko curves with different density-of-state effective masses (m*) are also plotted in Fig. 4c. Obviously, the increased *S* with decreasing *n* is attributed to the heavier m*. For the undoped GeTe, m* is 1.44 *m_e_* (where *m_e_* is the free electron mass) at 300 K. This increases to 2.04 *m_e_* for Ge_0.95_Mg_0.05_Te. Doping Sb into Ge_0.95_Mg_0.05_Te further increases m* to as high as 4.77 *m_e_*. The heavier m* in the (Mg, Sb) co-doped GeTe samples indicates that the carriers have lower velocities, being consistent with the measured lower *μ* in these samples (Fig. 4a and b). For comparison, the *S* and *n* data for the Ge_1-_*_x_*Sb*_x_*Te samples are also included in Fig. 4c [40,44]. Clearly, in the same *n* range, the *S* values for the Ge_0.95-_*_x_*Mg_0.05_Sb*_x_*Te samples are larger than those for the Ge_1-_*_x_*Sb*_x_*Te samples. In order to better understand the effect of Mg in GeTe, the electronic band structures and density-of-state (DOS) near the Fermi levels for both pure GeTe and Mg-doped GeTe rhombohedral supercells (3 × 3 × 3) are calculated. As shown in Fig. 4d and Supplementary Fig. S9, doping Mg significantly modifies the band structures of both the rhombohedral phase and cubic phase, yielding steeper DOS near the valence band edge, especially in the range from −0.1 to −0.3 eV. The steeper DOS leads to the higher m*, which is responsible for the larger *S* and lower *μ* values for the Ge_0.95-_*_x_*Mg_0.05_Sb*_x_*Te than those for the Ge_1-_*_x_*Sb*_x_*Te in a similar *n* range (Fig. 4b and d).

Generally, the weighted mobility *μ_W_* = *μ*(m*/*m_e_*)^3/2^ is a very effective performance indicator for the electrical transport in TE materials [45]. As shown in the inset of Fig. 4e, the *μ_W_* for Ge_0.95-_*_x_*Mg_0.05_Sb*_x_*Te at 300 K gradually increases with decreasing *n* due to the enhanced m*. Correspondingly, the *PF* values for Ge_0.95-_*_x_*Mg_0.05_Sb*_x_*Te exhibit similar *n*-dependence. As shown in Fig. 4e, Ge_0.85_Mg_0.05_Sb_0.1_Te has a maximum *PF* of 14.2 μW cm^−1 ^K^−2^ at 300 K, which is about three times that of Ge_0.95_Mg_0.05_Te. For comparison, Fig. 4e also plots the *PF* and *n* data for Ge_1-_*_x_*Sb*_x_*Te [40,44]. Despite the lower *μ* (Fig. 4b), the (Mg, Sb) co-doped GeTe samples possess similar *PF* values to those Sb single-doped Ge_1-_*_x_*Sb*_x_*Te in a similar *n* range due to their higher m*. However, it should be noted that the Ge_0.95-_*_x_*Mg_0.05_Sb*_x_*Te samples possess much lower *κ_L_* than the Ge_1-_*_x_*Sb*_x_*Te samples with the same Sb-doping content, which can be well demonstrated by Fig. 4f. The additional mass and strain field fluctuations introduced by the Mg dopants at Ge sites are responsible for this phenomenon. Finally, the well-maintained high *PF* and significantly lowered *κ_L_* in Ge_0.95-_*_x_*Mg_0.05_Sb*_x_*Te result in a higher *zT* than those for Ge_1-_*_x_*Sb*_x_*Te (Supplementary Fig. S10).

**Figure 5.**
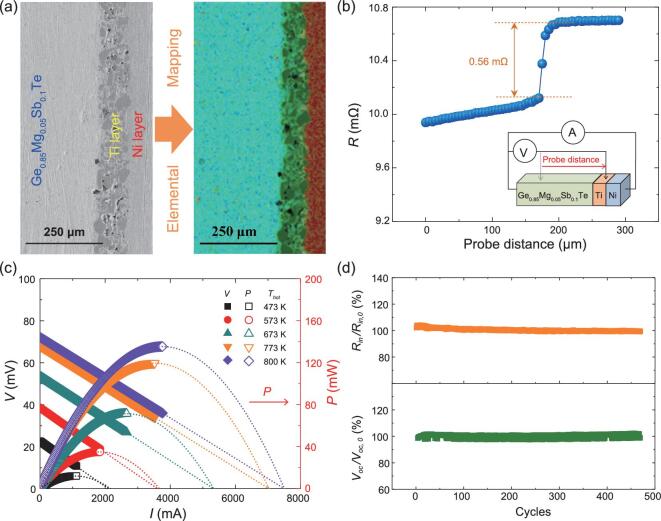
(a) Scanning electron microscopy (left panel) and EDS elemental mapping (right panel) performed on the interface area of the as-prepared Ni/Ti/Ge_0.85_Mg_0.05_Sb_0.1_Te uni-leg. (b) Resistance (*R*) line scanning across the Ni/Ti/Ge_0.85_Mg_0.05_Sb_0.1_Te interface for electrical contact resistivity (*R_c_*) measurement. The inset shows a schematic drawing of the *R_c_* measurement. (c) Output voltage (*V*) and power output (*P*) as a function of current (*I*) for the Ni/Ti/Ge_0.85_Mg_0.05_Sb_0.1_Te uni-leg under different operating temperatures. The cold side temperature of the uni-leg is 300 K. (d) Variations of relative internal resistance (*R*_in_/*R*_in_*_,0_*) and relative open circuit voltage (*V*_oc_/*V*_oc_*_,0_*) for the Ni/Ti/Ge_0.85_Mg_0.05_Sb_0.1_Te uni-leg during the thermal cycling test, where *R*_in_*_,0_* and *V*_oc_*_,0_* are the initial internal resistance and initial open circuit voltage, respectively. The data are collected when the hot side temperature of the uni-leg is 800 K.

The Ge_0.95-_*_x_*Mg_0.05_Sb*_x_*Te in this study provides a good possibility to fabricate stable and efficient TE modules applied in the mid-temperature range. Here, a GeTe-based TE uni-leg is obtained by one-step sintering of the Ge_0.85_Mg_0.05_Sb_0.1_Te powder, Ti powder, and Ni powder directly. The dimensions of the Ni/Ti/Ge_0.85_Mg_0.05_Sb_0.1_Te uni-leg are 4 × 6 × 8 mm. Excellent bonding without any cracks is observed in the Ti/Ge_0.85_Mg_0.05_Sb_0.1_Te interface area and Ni/Ti interface area (Fig. 5a). Furthermore, backscatter electron imaging mapping shows that there is no obvious diffusion layer formed near the interface areas (Fig. 5a). The electrical contact resistivity (*R_c_*) of the Ti/Ge_0.85_Mg_0.05_Sb_0.1_Te interface in the uni-leg is measured on a home-made instrument [46]. As shown in Fig. 5b, a leap is observed from the Ge_0.85_Mg_0.05_Sb_0.1_Te side to the Ti side. The calculated *R_c_* based on this leap is 134 μΩ cm^2^, which is much larger than those observed in other TE modules, such as 20 μΩ cm^2^ for a CoSb_3_/Ti interface [47]. However, the electrical contact resistance caused by the *R_c_* only contributes about 5% of the total resistance of the Ni/Ti/Ge_0.85_Mg_0.05_Sb_0.1_Te uni-leg; thus it is still an acceptable value.

The performance of the Ni/Ti/Ge_0.85_Mg_0.05_Sb_0.1_Te TE uni-leg is measured using a home-built testing system. The current, output voltage, power output, and internal resistance are measured under different operating temperatures. Figure 5c shows the *I*–*V* curves, which exhibit a good linear relationship. The maximum power output (*P*_max_) is 135 mW with the hot side temperature (*T*_hot_) of the uni-leg being 800 K and the temperature difference (*ΔT*) across the uni-leg being 500 K. If the electrical contact resistivity *R_c_* of the Ti/Ge_0.85_Mg_0.05_Sb_0.1_Te interface can be further reduced, higher *P*_max_ is expected.

A thermal cycling test is performed on the Ni/Ti/Ge_0.85_Mg_0.05_Sb_0.1_Te TE uni-leg to confirm its service stability. The hot side temperature is cycled between 473 K and 800 K, while the cold side temperature is fixed at 300 K. The output voltage, internal resistance, and power output of the uni-leg are collected when the hot side temperature is raised to 800 K. Normally, if any cracks have formed inside the material or at the interface area, the resistance will greatly increase, which would lead to a significant deterioration of the power output [48]. As shown in Fig. 5d and Fig. 1d, the internal resistance, output voltage, and power output are quite stable after even 450 cycles, suggesting that the Ge_0.85_Mg_0.05_Sb_0.1_Te material and its interface are well maintained during the thermal cycling test. This can be further confirmed by the scanning electron microscopy (SEM) characterization performed on the Ni/Ti/Ge_0.85_Mg_0.05_Sb_0.1_Te TE uni-leg after the thermal cycling test (Supplementary Fig. S11), which shows no cracks inside the material or near the interface area. The suppressed volume variation during the rhombohedral–cubic phase transition due to the improved CTE match between the rhombohedral and cubic phases (Fig. 1c) is believed to be responsible for the high service stability of the present Ni/Ti/Ge_0.85_Mg_0.05_Sb_0.1_Te TE uni-leg.

## CONCLUSIONS

In summary, this study demonstrates that co-doping Mg and Sb into GeTe can obtain stable and efficient mid-temperature TE materials. A maximum *zT* of 1.84 at 800 K and a high average *zT* of 1.2 at 300–800 K have been achieved by simultaneously reducing lattice thermal conductivity and optimizing carrier concentration. More importantly, the suppressed volume variation during the phase transition due to the improved CTE match between the rhombohedral and cubic phases ensures that the TE uni-leg made by Ge_0.85_Mg_0.05_Sb_0.1_Te possesses quite high service stability even after 450 thermal cycles. This work should promote the real application of GeTe-based materials for TE power generation in the mid-temperature range.

## METHODS

### Materials synthesis

High-quality polycrystalline samples were synthesized by melting of stoichiometric ratios of pure elemental Ge (shots, 99.999%), Te (shots, 99.999%), Sb (shots, 99.999%), and Mg (shots, 99.9%) in evacuated and sealed silica tubes. The mixtures were slowly heated up to 1373 K over 11 h and soaked at this temperature for 12 h, then quenched in ice water, followed by annealing at 873 K for five days. Next, the obtained ingots were hand-ground into fine powders in an agate mortar. Spark plasma sintering (SPS, Dr Sinter: SPS-2040) was carried out to obtain dense bulk samples under a uniaxial pressure of 60 MPa at 823 K for 10 min in graphite dies. The densities of the pellets were > 98% of the theoretical values, which are listed in Table 2.

### Material characterization

Powder X-ray diffraction (D8 Advance, Bruker) was performed to analyze the phase purity of samples using Cu Kα radiation (λ = 1.5406 Å) at ambient temperature. The lattice parameters were refined with a least-squares refinement method utilizing the WinCSD program package. The microstructures were observed by scanning electron microscopy (SEM, ZEISS Supra 55) and the chemical compositions were characterized using energy dispersive spectrometry (EDS). A field emission transmission electron microscope (TEM, JEM-2100F) was employed to identify the crystal structure of the prepared samples. The electrical conductivity and Seebeck coefficient were measured on bulk samples with approximate dimensions of 2 × 2 × 8 mm, using commercial equipment (ZEM-3, ULVAC) under He atmosphere from 300 to 800 K. The thermal conductivity was calculated by *κ* = *D* × *C_p_* × *ρ*, where the heat capacity (*C_p_*) was estimated using the Dulong–Petit law, the thermal diffusivity (*D*) was measured by the laser flash system (LFA457, Netzsch) under argon atmosphere and the density (*ρ*) was obtained using the Archimedes method. Hall coefficients (*R_H_*), electrical conductivity and thermal conductivity from 5 K to 300 K were measured by a physical property measurement system (PPMS, Quantum Design). The maximum magnetic field reached 5 T in both positive and negative directions. The carrier concentration *p* and the carrier mobility (*μ*) were calculated according to the relation *p* = 1/*eR_H_* and *μ* = *σR_H_*, respectively. Differential scanning calorimetric measurements (Netzsch DSC 404F3) were employed with a heating rate of 20 K/min to determine the phase transition characters of samples. The relative length variation (*dL*/*L_0_*) with increasing temperature was measured by thermal expansion equipment (Netzsch, DIL 402 C). The linear coefficient of thermal expansion (CTE) was obtained by dividing the *dL*/*L_0_* in the assigned temperature range by the temperature difference (Δ*T*).

### Uni-leg TE fabrication and testing

The p-type Ge_0.85_Mg_0.05_Sb_0.1_Te TE uni-leg with a thin Ti film as barrier layer and a thin Ni film as electrode was fabricated by sintering Ge_0.85_Mg_0.05_Sb_0.1_Te, Ti powder, and Ni powder directly using the hot-pressing technique. The sintering temperature, press, and time are 873 K, 60 MPa, and two hours, respectively. The obtained bulk was cut into bars with designed geometry using wire cutting. The Ni/Ti/Ge_0.85_Mg_0.05_Sb_0.1_Te interface was characterized by field emission scanning electron microscopy (FESEM, Magellan-400). The room-temperature electrical contact resistivity of the Ni/Ti/Ge_0.85_Mg_0.05_Sb_0.1_Te interface was measured by a home-made four-probe platform with the measurement details shown in Ref. [46]. The geometry of the measured sample is 4 × 6 × 8 mm^3^. The performance of the Ni/Ti/Ge_0.85_Mg_0.05_Sb_0.1_Te uni-leg is elevated by a home-made instrument. A schematic drawing is shown in Supplementary Fig. S12. The current, output voltage, internal resistance, and power output were recorded at the temperature ramping process when the hot side temperature of the uni-leg reached 473 K, 573 K, 673 K, 773 K and 800 K. Circulating water with a fixed temperature of 300 K was used to cool the cold side. The maximum power output was obtained when the external electrical load was equal to the uni-leg's internal resistance. A thermal cycling test was performed by using a home-built testing system. The hot side temperature was repeatedly heated to 800 K at a rate of 50 K/min and held at this temperature for 5 min. Then the temperature was cooled to 473 K in 15 min. The output voltage, internal resistance, and power output of Ni/Ti/Ge_0.85_Mg_0.05_Sb_0.1_Te were collected when the hot side temperature was cycled to 800 K.

### Calculation of the band structure

The first-principles calculations for the band structures were performed by employing the Vienna *Ab Initio* Simulation Package (VASP), implemented with the generalized gradient approximation functional and the projector-augmented wave (PAW) method. A 3 × 3 × 3 supercell of the GeTe formula unit was constructed for both the GeTe and Mg-doped GeTe with a cut-off energy of 400 eV for the plane-wave and an energy convergence criterion of 10^−3^ eV per unit cell. The configuration with the lowest energy, optimized lattice parameters and atom positions is used for the defect-containing supercell.

## Supplementary Material

nwz052_Supplemental_File
