# Supplementary material for: Superior performance and high service stability for GeTe-based thermoelectric compounds
Source: Natl Sci Rev. 2019 Apr 10;6(5):944–54. doi: 10.1093/nsr/nwz052 (PMC8291431; doi:10.1093/nsr/nwz052)
Supplement: nwz052_Supplemental_File [file nwz052_supplemental_file.docx]

**Supplementary Information**

**Superior performance and high service stability for GeTe-based thermoelectric compounds**

*Tong Xing^1,2#^, Qingfeng Song ^1,2#^,* *Pengfei Qiu^1^, Qihao Zhang^1^, Xugui Xia^1^, Jincheng Liao^1^, Ruiheng Liu^1^, Hui Huang^1^, Jiong Yang^3^, Shengqiang Bai^1^, Dudi Ren^1^, Xun Shi^1*^, Lidong Chen^1*^*

^1^State Key Laboratory of High Performance Ceramics and Superfine Microstructure, Shanghai Institute of Ceramics, Chinese Academy of Sciences, Shanghai 200050, China

^2^Center of Materials Science and Optoelectronics Engineering, University of Chinese Academy of Sciences, Beijing 100049, China

^3^Materials Genome Institute, Shanghai University, Shanghai, 200444, China

#The two authors contribute equally to this work.

Corresponding author: [xshi@mail.sic.ac.cn](mailto:xshi@mail.sic.ac.cn); [cld@mail.sic.ac.cn](mailto:cld@mail.sic.ac.cn)


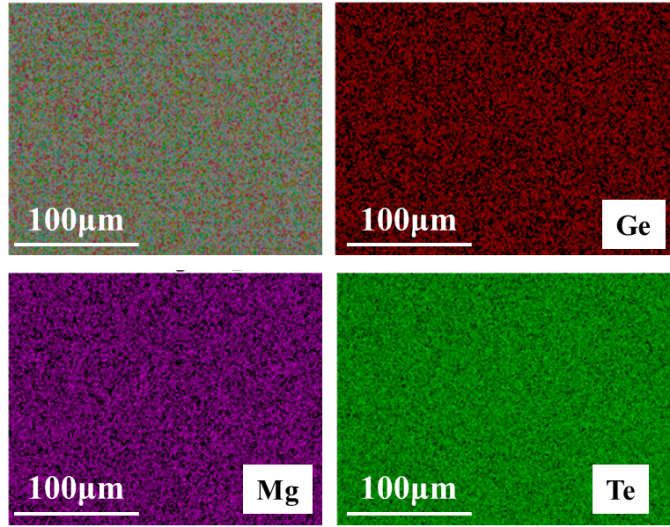


Figure S1 Elemental distribution mappings for Ge_0.95_Mg_0.05_Te.


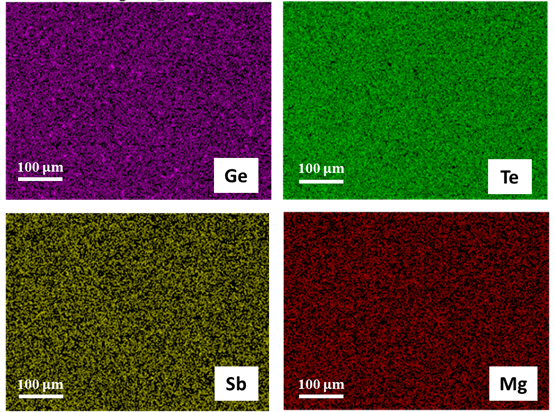


Figure S2 Elemental distribution mappings for Ge_0.85_Mg_0.05_Sb_0.1_Te.


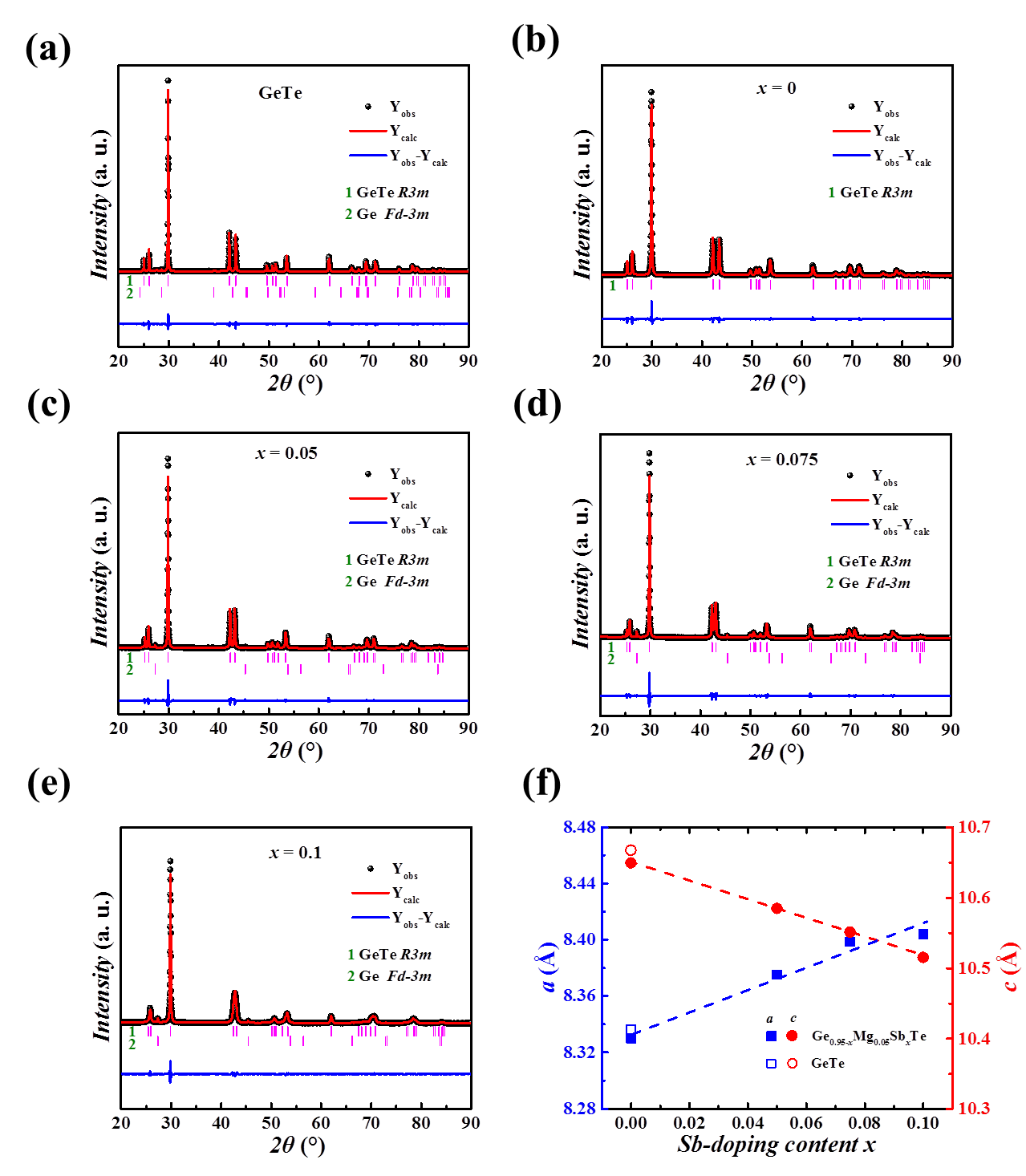


Figure S3 Refined power XRD patterns and lattice parameters of rhombohedral structure (*R3m*) for Ge_0.95-_*_x_*Mg_0.05_Sb*_x_*Te (*x* = 0, 0.05, 0.075, and 0.1).


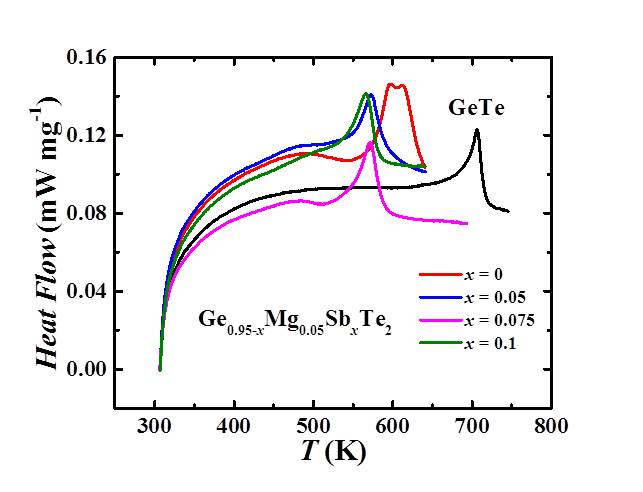


Figure S4 Heat flow curves of Ge_0.95-x_Mg_0.05_Sb*_x_*Te (*x* = 0, 0.05, 0.075, and 0.1).


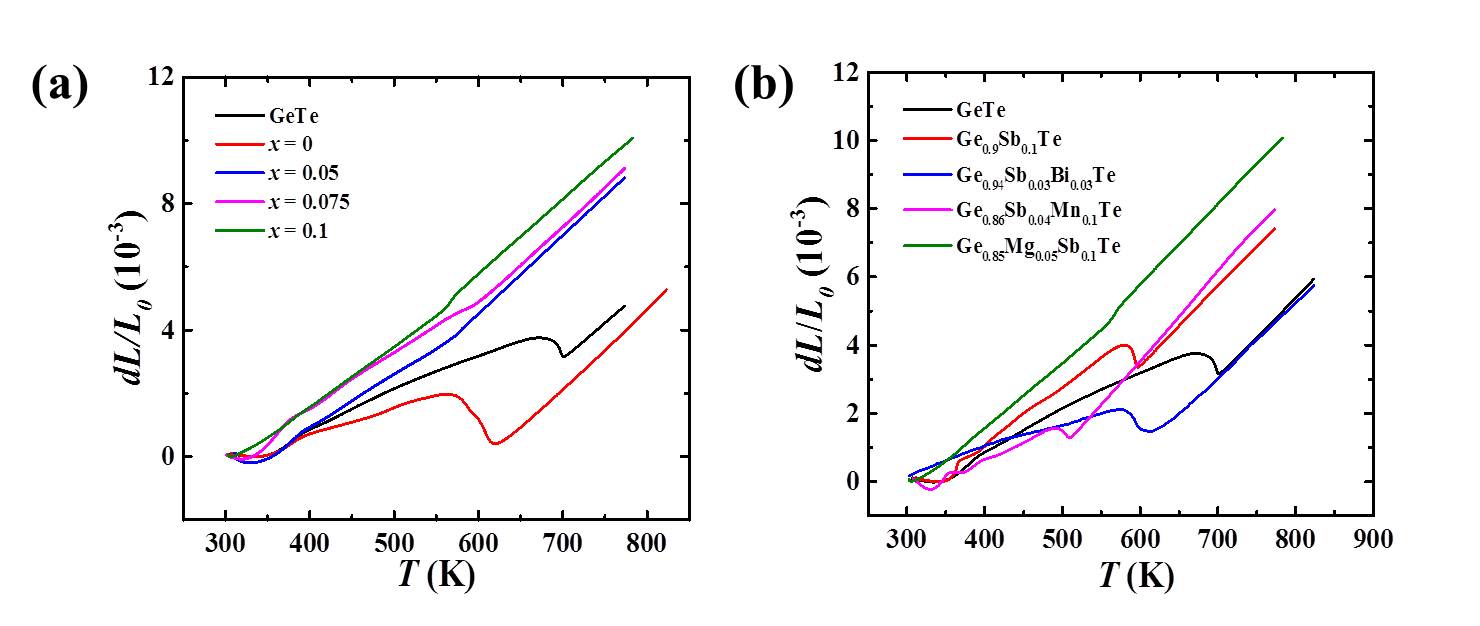


Figure S5 Temperature dependence of relative length variation (*dL*/*L_0_*) for (a) Ge_0.95-_*_x_*Mg_0.05_Sb*_x_*Te (*x* = 0, 0.05, 0.075, and 0.1) and (b) some typical GeTe-based materials reported in literatures.


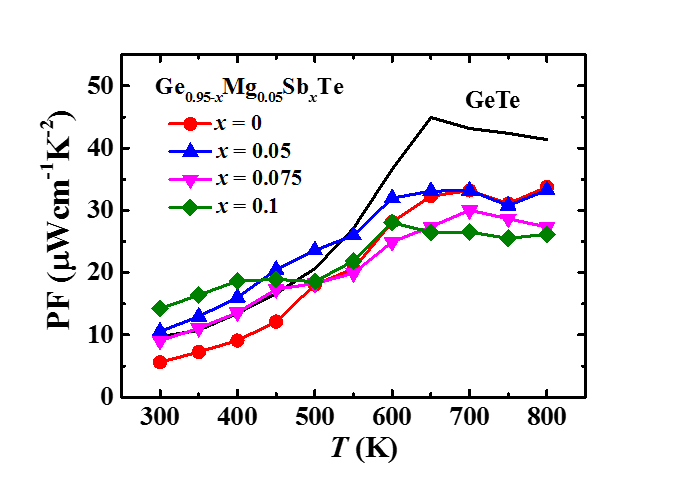


Figure S6 Temperature dependence of power factor (*PF*) for Ge_0.95-_*_x_*Mg_0.05_Sb*_x_*Te (*x* = 0, 0.05, 0.075, and 0.1).


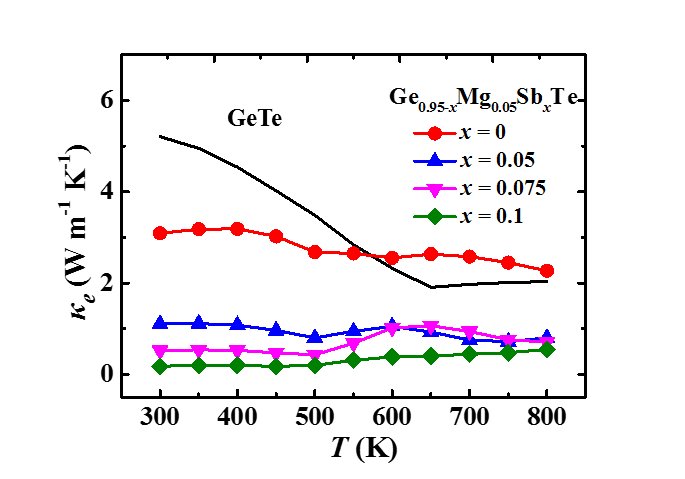


Figure S7 Temperature dependence of the calculated carrier thermal conductivity (*κ_e_*) for Ge_0.95-_*_x_*Mg_0.05_Sb*_x_*Te (*x* = 0, 0.05, 0.075, and 0.1).


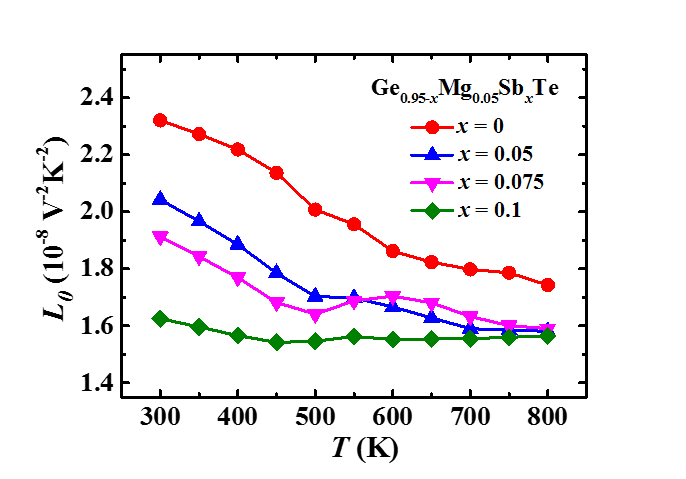


Figure S8 Temperature dependence of the calculated Lorenz number (*L*_0_) for Ge_0.95-_*_x_*Mg_0.05_Sb*_x_*Te (*x* = 0, 0.05, 0.075, and 0.1).


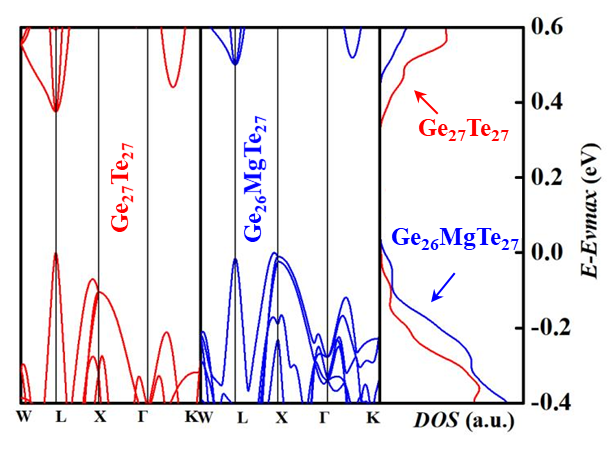


Figure S9 Calculated band structure and density-of-state near the Fermi level for both the un-doped GeTe and Mg-doped GeTe cubic supercell.


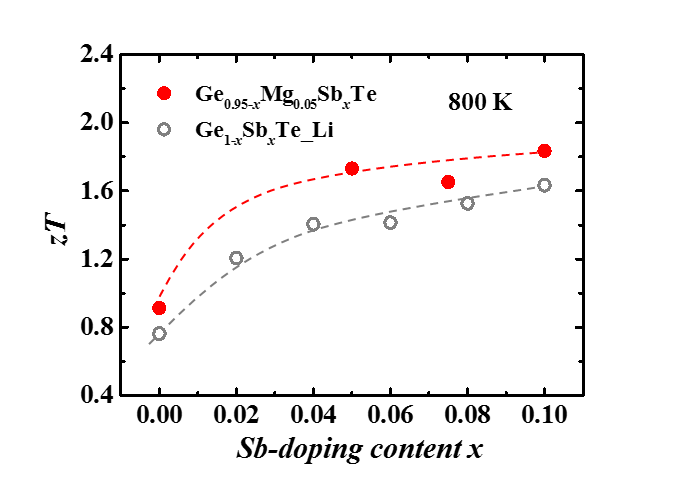


Figure S10 TE figure of merit (*zT*) as a function of Sb-doping content *x* at 800 K. The data for the Ge_1-_*_x_*Sb*_x_*Te samples are included for comparison. The dashed lines are guides to the eyes.


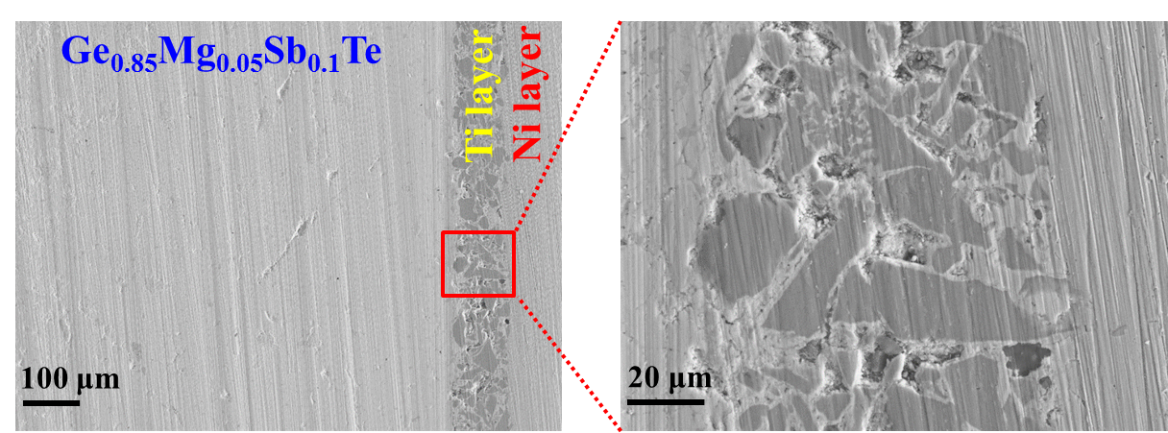


Figure S11 Scanning electron images of the Ni/Ti/Ge_0.85_Mg_0.05_Sb_0.1_Te interface after experiencing 450 times thermal cycles.


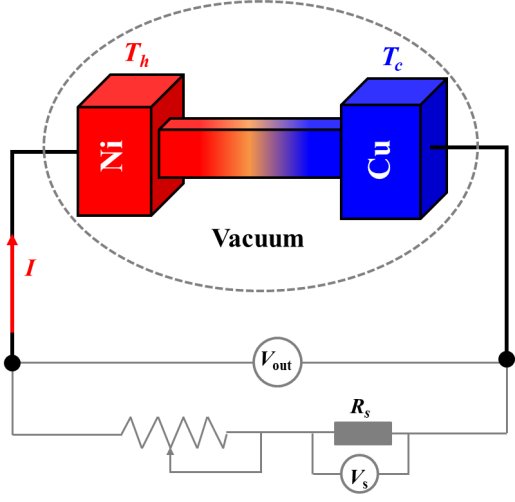


Figure S12 Schematic drawing of the TE uni-leg test instrument.
